# Effect of Dietary Nitrogen-to-Sulfur Ratio on Rumen Microbiota, Metabolites, and Ruminal Antioxidant Status in Tibetan Sheep

**DOI:** 10.3390/antiox15080913

**Published:** 2026-07-23

**Authors:** Zhendong Liu, Minglan He, Wenling Xie, Shengzhen Hou, Lijuan Han, Chao Yang, Zhenzhen Yuan, Linsheng Gui, Shengnan Sun

**Affiliations:** College of Agriculture and Animal Husbandry, Qinghai University, Xining 810016, China; ys250951331513@qhu.edu.cn (Z.L.); ys250955001619@qhu.edu.cn (M.H.); ys240951350700@qhu.edu.cn (W.X.); 1987990009@qhu.edu.cn (S.H.); 2016990034@qhu.edu.cn (L.H.); yangchao@qhu.edu.cn (C.Y.); 2017990038@qhu.edu.cn (Z.Y.); 2017990039@qhu.edu.cn (L.G.)

**Keywords:** nitrogen–sulfur ratio, antioxidant, volatile fatty acids, rumen microbiota, untargeted metabolomics

## Abstract

Optimizing the dietary nitrogen-to-sulfur (N:S) ratio is crucial for enhancing ruminal microbial fermentation and nutrient digestibility. This study investigated the effects of different dietary N:S ratios on ruminal antioxidant indices, digestive enzyme activities, immune parameters, volatile fatty acid (VFA) profiles, microbial communities, and metabolomic profiles in plateau-type Tibetan sheep. Ninety 2-month-old male Tibetan sheep (initial body weight, 15.55 ± 0.20 kg) were randomly assigned to three dietary treatments formulated with N:S ratios of 9.5:1 (HP-H), 8.5:1 (HP-M), and 7.5:1 (HP-L). Sheep fed the HP-H diet exhibited improved ruminal antioxidant indices compared with the other treatments, with CAT and T-AOC activities being higher than those of both the HP-M and HP-L groups, while SOD and GSH-Px activities were significantly higher only compared with those in the HP-L *(p* < 0.05). Furthermore, ruminal cellulase activity, as well as propionate and butyrate levels, were significantly higher in the HP-H group (*p* < 0.05). Microbiome analysis revealed that the HP-H diet enriched the relative abundances of *Candidatus Saccharimonas*, *the Rikenellaceae RC9 gut group*, and *uncultured rumen bacteria*. Correspondingly, untargeted metabolomics identified higher signal intensities of 3-hydroxyphenylacetic acid, nervonic acid, arachidonic acid (peroxide-free), and calciferol in the ruminal fluid of the HP-H group relative to the other treatments. In conclusion, when the nitrogen-to-sulfur ratio was 9.5:1, ruminal antioxidant enzyme activities were increased, and the concentrations of propionic acid and butyric acid were increased. The differential microorganisms and metabolites were mainly associated with lipid metabolism, as well as cofactor and vitamin metabolism.

## 1. Introduction

Tibetan sheep (*Ovis aries*) are a unique livestock breed and a valuable germplasm resource indigenous to the Qinghai–Tibet Plateau. Endemic species in this region have evolved to survive under extreme environmental stressors, including high altitude, severe cold, hypoxia, and intense ultraviolet radiation [1]. Through long-term natural and artificial selection, Tibetan sheep have developed remarkable physiological characteristics, particularly cold tolerance and high-altitude adaptability [2]. The rumen, a defining physiological feature of ruminants, is crucial for nutrient utilization, metabolic regulation, and overall host health [3]. Harboring a complex microbial ecosystem comprising bacteria, archaea, fungi, and protozoa, the rumen ferments dietary carbohydrates and proteins into short-chain fatty acids (SCFAs), microbial protein, and ammonia [4]. SCFAs serve as the primary energy source for the host, while microbial protein supplies essential amino acids [5]. Although Tibetan sheep exhibit robust environmental adaptability, the underlying mechanisms governing ruminal metabolic regulation and microbial adaptation remain incompletely elucidated [6].

Nitrogen and sulfur are indispensable nutrients for protein synthesis and microbial metabolism in ruminants [7,8]. Nitrogen serves as a primary constituent of amino acids and nucleic acids, whereas sulfur is essential for the synthesis of sulfur-containing amino acids (such as methionine and cysteine), vitamins, and coenzymes [9]. These two elements are metabolically intertwined within the ruminal microbiome [10]. Previous studies have demonstrated that maintaining an appropriate balance between dietary nitrogen and sulfur is essential for optimizing rumen microbial protein synthesis, nutrient utilization, and animal performance in ruminants [11]. However, excessive nitrogen intake decreases utilization efficiency and exacerbates nitrogen excretion, while an imbalanced N:S ratio restricts microbial activity and fermentation capacity [12]. Previous studies indicate that the dietary N:S ratio significantly influences nutrient digestibility, production performance, and wool growth in ruminants [13]. Despite its importance, most research has focused on conventional sheep and goat breeds, leaving a critical knowledge gap regarding Tibetan sheep managed under high-altitude conditions.

The integration of 16S rRNA sequencing and untargeted metabolomics offers a robust approach to simultaneously profile the ruminal microbial community and its metabolic outputs, providing new opportunities to elucidate their coordinated regulation [14]. Therefore, this study aimed to investigate the effects of varying dietary N:S ratios on ruminal antioxidant capacity, microbial community dynamics, and metabolomic profiles in Tibetan sheep. Our findings provide novel insights into the nutritional regulation of plateau-dwelling ruminants and establish a theoretical framework for optimizing high-altitude feeding strategies.

## 2. Materials and Methods

### 2.1. Experimental Design and Sample Collection

The animal feeding trial was conducted at Jinzang Animal Husbandry Company (Xihai Town, Haiyan County, Qinghai Province, China). Ninety lambs (2-month-old, initial body weight 15.55 ± 0.20 kg) were randomly allocated to 18 pens, with six pens per treatment and five lambs per pen. Three nitrogen-to-sulfur (N:S) ratios were set as 9.5:1 (HP-H), 8.5:1 (HP-M), and 7.5:1 (HP-L). The trial lasted for 100 days, comprising a 10-day adaptation period and a 90-day experimental period. The ingredients and nutritional composition of the experimental diets are detailed in Table 1. The concentration and roughage were thoroughly mixed (7:3, dry matter) and offered twice daily (at 08:00 and 16:30), with all animals having ad libitum access to feed and water.

At the end of the experiment, after a 12 h overnight fasting period, one lamb close to the average body weight was selected from each pen and slaughtered at a commercial slaughterhouse. Following slaughter, the abdominal cavity was opened, and the rumen was immediately isolated. Ruminal fluids were strained through four layers of sterile cheesecloth and aliquoted into 10 mL sterile cryovials (Corning Inc., Corning, NY, USA; Cat. No. SCT-012-500-C-S). The digestive enzyme activities, immune indices, and volatile fatty acid (VFA), microbe and metabolite of ruminal fluid were determined.

Approximately 2–4 cm^2^ of ruminal tissue was collected from a consistent anatomical location of the ventral sac and rapidly fixed in 4% paraformaldehyde (Leagene Biotechnology Co., Ltd., Beijing, China; Cat. No. DF0135) for phenotypic determination. Subsequently, the ruminal tissue, about 3 to 4 g, was packaged in a 5 μL Eppendorf tube (Solarbio, Beijing, China) and then stored at −80 °C for antioxidant capacity analysis.

### 2.2. Antioxidant Indicator Assay in Rumen Tissue

The antioxidant indicators, including total antioxidant capacity activity (T-AOC; A015-2-1), superoxide dismutase activity (SOD; A001-3-2), catalase activity (CAT; A007-1-1), glutathione peroxidase activity (GSH-Px; A005-1-2) and malondialdehyde concentration (MDA; A003-1-2), were determined using commercial kits (Nanjing Jiancheng Bioengineering Institute, Nanjing, China) according to the manufacturer’s instructions. Briefly, approximately1 g of rumen tissue was mixed with 9 mL of phosphate-buffered saline (PBS, Shanghai Jingshen Technology Co., Ltd., Shanghai, China) and homogenized. The homogenate was centrifuged at 3000× *g* for 15 min at 4 °C. The blank well received 50 μL of sample diluent. The standard wells received 50 μL of serially diluted standards, and the sample wells received 50 μL of the test sample. Except for the blank well, 50 μL of the corresponding detection antibody was added to each well. The plate was covered and incubated at 37 °C for 60 min. After incubation, the liquid in each well was discarded, and 350 μL of wash solution was added. After 30 s, the wash solution was removed, and the washing step was repeated five times. The plate was then patted dry. Then, 50 μL of substrate solution was added to each well, and the plate was incubated at 37 °C for 15 min in the dark. After incubation, 50 μL of stop solution (2 M H_2_SO_4_) was added to each well to terminate the reaction. The absorbance (OD value) at 450 nm was measured using a microplate reader (BioTek Instruments, Winooski, VT, USA). A standard curve was independently established for each microplate, and the correlation coefficient (R^2^) of the standard curve was required to be greater than 0.95.

### 2.3. Determination of Digestive Enzyme Activity and Immune Indexes

The activities of α-amylase, trypsin, chymotrypsin, cellulase, and lipase, as well as the concentrations of immunoglobulins (IgA, IgG, and IgM) in the ruminal fluid, were quantified using commercial enzyme-linked immunosorbent assay (ELISA) kits (Shanghai Meilian Biotechnology Co., Ltd., Shanghai, China). Absorbance values were recorded utilizing a multifunctional microplate reader (Thermo Fisher Scientific, Waltham, MA, USA). All analytical procedures were conducted in strict accordance with the manufacturer’s protocols. The specific kits used were as follows: α-amylase (YJ340341B), chymotrypsin (YJ343430B), cellulase (YJ234301B), trypsin (YJ6007096), lipase (YJ6009096), IgM (YJ074038), IgA (YJ074033), and IgG (YJ061717). Samples were appropriately diluted with assay buffer prior to analysis to ensure that absorbance values fell within the linear range of the standard curves. All samples were analyzed in duplicate, and intra- and inter-assay coefficients of variation were maintained below 10% to ensure analytical reliability.

### 2.4. Determination of Rumen Volatile Fatty Acid Concentration

Ruminal fluid was centrifuged (12,000× *g*, 10 min, 4 °C; Eppendorf 5810R) and the supernatant filtered through a 0.22 μm membrane. An aliquot was acidified with 25% metaphosphoric acid. VFAs (acetate, propionate, butyrate, valerate) were quantified by HPLC-UV (Waters-Baseline 520, UV detector) on a C18 reversed-phase column (4.6 × 250 mm, 5 μm) at 30 °C, with isocratic elution using 0.02 M KH_2_PO_4_/H_3_PO_4_ buffer (pH 2.37) at 1.0 mL/min, 10 μL injection, and detection at 214 nm. External calibration used acetic, propionic, butyric, and valeric acid standards (R^2^ > 0.999). Ammonium chloride was employed only as a system suitability standard, not for VFA quantification. Ammonia nitrogen was determined independently by the phenol-hypochlorite colorimetric method (Berthelot reaction). The supernatant was reacted with phenol, alkaline hypochlorite, and sodium nitroprusside, incubated in a water bath (37 °C, 20 min), and absorbance was measured at 630 nm using a microplate reader (BioTek EL × 808). Calibration was performed with ammonium sulfate standards (not ammonium chloride). All analyses were conducted by Shanghai Zhongke Testing Technology Co., Ltd. (Shanghai, China).

### 2.5. 16S rRNA Gene Amplicon Analysis

#### 2.5.1. Genomic DNA Extraction and High-Throughput Sequencing

Extraction and high-throughput sequencing. Total ruminal genomic DNA was extracted utilizing a magnetic bead-based genomic DNA extraction kit (DP712, Tiangen Biotech, Beijing, China), and its integrity was verified via 1% agarose gel electrophoresis. The V3-V4 hypervariable region of the bacterial 16S rRNA gene was amplified using the primer pair 341F (5′-CCTAYGGGRBGCASCAG-3′) and 806R (5′-GGACTACNNGGGTATCTAAT-3′), formulated with Phusion^®^ High-Fidelity PCR Master Mix (New England Biolabs, Ipswich, MA, USA). The thermocycling protocol consisted of an initial denaturation at 98 °C for 1 min, followed by 30 cycles of denaturation at 98 °C for 10 s, annealing at 50 °C for 30 s, and extension at 72 °C for 30 s, with a final extension step at 72 °C for 5 min. Amplicons with a target size of 400–450 bp were resolved by 2% agarose gel electrophoresis, excised, and purified using a QIAquick Gel Extraction Kit (Qiagen, Hilden, Germany). Sequencing libraries were constructed utilizing the TruSeq^®^ DNA PCR-Free Sample Preparation Kit (Illumina, San Diego, CA, USA) according to the manufacturer’s recommendations. Following fluorometric quantification (Qubit 2.0 Fluorometer, Thermo Fisher Scientific) and quality assessment (Agilent 2100 Bioanalyzer, Agilent Technologies, Santa Clara, CA, USA), the libraries were sequenced on an Illumina NovaSeq 6000 platform to generate 250 bp paired-end reads (Shanghai Zhongke New Life Biotechnology Co., Ltd., Shanghai, China). The sequencing depth was normalized to 44,000 reads per sample for downstream analyses. Raw FASTQ sequencing data were demultiplexed by barcode using FLASH software. Raw reads were quality-filtered by removing reads containing ambiguous bases, low-quality reads (Q-score < 20), and sequences shorter than the threshold length. The number of raw reads per sample ranged from approximately 59,000 to 86,000, while clean reads ranged from approximately 56,000 to 84,000. After quality filtering and chimera removal, effective reads ranged from approximately 44,000 to 66,000 per sample.

#### 2.5.2. Bioinformatics Analysis Process

Paired-end reads were merged using FLASH software (version 1.2.11), and demultiplexing was performed based on barcode information prior to quality filtering. Chimeric sequences were identified and removed using the UCHIME algorithm to ensure data quality for downstream analysis. Subsequently, operational taxonomic units (OTUs) were clustered at a 97% sequence similarity threshold using the UPARSE algorithm. Although amplicon sequence variant (ASV)-based approaches are currently considered more precise, an OTU-based (97% similarity) clustering strategy was adopted in this study for consistency with previous rumen microbiome studies. Representative sequences for each OTU were aligned against the SILVA database (Release 132) using the RDP Classifier for taxonomic annotation. Following the rarefaction of sequence data to a uniform depth across all samples, alpha-diversity metrics (Chao1, observed species, Shannon, and Simpson indices) were calculated, and rarefaction curves were generated. Good’s coverage values (>0.99) indicated that sequencing depth was sufficient to capture the majority of bacterial diversity. Taxonomic composition was dynamically visualized using Krona, and the distribution of taxa was represented via Venn diagrams. Non-metric multidimensional scaling (NMDS) was employed for beta-diversity dimensional reduction analysis. Finally, linear discriminant analysis effect size (LEfSe) was utilized to identify taxonomic biomarkers exhibiting statistically significant differential abundances among the dietary groups. No negative control samples were included in the present study; therefore, potential environmental or reagent contamination cannot be completely excluded. The datasets presented in this study can be found in the NCBI Sequence Read Archive (SRA) under accession number PRJNA1472923.

### 2.6. Non-Targeted Metabolomics Analysis

#### 2.6.1. Metabolite Extraction

Ruminal fluid samples were collected from each animal and immediately centrifuged to remove solid particles. The supernatant was stored at −80 °C until analysis. Metabolites were extracted using pre-cooled methanol/acetonitrile (1:1, *v*/*v*), followed by vortex mixing and ice-bath incubation. Samples were centrifuged at 14,000× *g* for 15 min at 4 °C, and the supernatant was collected for LC-MS analysis.

#### 2.6.2. Liquid Chromatography-Mass Spectrometry (LC-MS) Analysis

Liquid chromatography-mass spectrometry (LC-MS) analysis was performed using an ACQUITY UPLC BEH Amide chromatographic column (2.1 mm × 100 mm, 1.7 μm) for hydrophilic interaction chromatography (HILIC) separation. All samples were analyzed in a randomized order to minimize analytical bias. Mobile phase A was an aqueous solution containing 25 mM ammonium acetate and 25 mM ammonium hydroxide, and mobile phase B was acetonitrile. The gradient program was as follows: 85% B was maintained from 0 to 1 min, decreased linearly to 65% B from 1 to 12 min, suddenly decreased to 40% B from 12 to 12.1 min and was maintained until 16.1 min, before increasing to 85% B from 16.1 to 16.2 min, followed by a 5 min equilibration. The flow rate was 0.4 mL/min, the column temperature was 25 °C, the injection volume was 2 μL, and the autosampler temperature was 5 °C. Mass spectrometry detection was carried out using a Sciex TripleTOF 6600 quadrupole (SCIEX, Framingham, MA, USA) time-of-flight mass tandem spectrometer. The electrospray ionization source (ESI) was operated in both positive and negative modes. The source parameters were Gas1/Gas2 = 60, CUR = 30, source temperature 600 °C, and spray voltage ±5500 V. The primary scan range was *m/z* 60–1000 with an acquisition time of 0.20 s/spectrum. The secondary scan used the information-dependent acquisition (IDA) high-sensitivity mode with a range of *m/z* 25–1000, an acquisition time of 0.05 s/spectrum, a fixed collision energy of 35 ± 15 eV, a declustering voltage of ±60 V, 10 candidate ions were monitored per cycle, and isotope peaks within 4 Da were excluded.

Raw LC-MS data were converted to mzXML format using ProteoWizard software (V3.0.20287). The peak detection, retention time alignment, normalization, and deconvolution were performed using the XCMS package (V4.40) with standard parameter settings. Metabolite annotation was performed by matching accurate mass, MS/MS fragmentation patterns, isotope distributions, and retention time against entries in public databases (HMDB and METLIN). Compounds were assigned confidence levels based on predefined annotation scoring criteria. Features with >50% missing values within a group were removed, and remaining missing values were imputed with half the minimum non-zero intensity across the data to generate a quantitative data matrix for downstream analyses.

The multivariate statistical analyses, including the principal component analysis (PCA) and the partial least squares discrimination analysis (PLS-DA), were carried out using the ropls package (version 1.6.2, R software). The differential metabolites between groups were identified based on the variable importance (VIP) from the OPLS-DA and Student’s *t*-test (VIP >1 and *p* < 0.05). The fold-change (FC) value of each metabolite was calculated by comparing the means of the peak area, and the log2FC value was used to indicate the specific variable quantity in the comparison. Additionally, metabolic pathway analysis was performed according to the Kyoto Encyclopedia of Genes and Genomes (KEGG, http://www.kegg.jp) to reveal the biological importance of the metabolites. Pathway-level differences between each treatment and the control were evaluated using two-sided Welch’s *t*-tests, with Benjamini–Hochberg false discovery rate (FDR) correction (FDR < 0.05).

### 2.7. Statistical Analysis

Besides the microbial and the untargeted metabolomics data, the phenotypic data were analyzed using SPSS version 26.0 software package (IBM Corp., Armonk, NY, USA). The data are reported as means ± SEM. Levene’s test of homogeneity of variance was used to evaluate unequal variances before analysis of variance (ANOVA). Data were analyzed by 1-way ANOVA, and Duncan’s multiple range test was used to examine differences between the means. The statistical significance threshold was set at *p* < 0.05. The statistical analysis and image presentation were performed in GraphPad Prism (version 8.0).

Correlation analysis among key variables was performed using Pearson’s correlation coefficient. To reduce false-positive associations, only correlations with |r| > 0.60 and *p* < 0.05 were considered biologically relevant. A unified statistical workflow was applied to integrate microbiome, metabolomics, and phenotypic datasets for multi-omics analysis.

## 3. Results

### 3.1. Antioxidant Index

As shown in Table 2, catalase (CAT) and total antioxidant capacity (T-AOC) activities were significantly higher in the HP-H group than in both the HP-M and HP-L groups (*p* < 0.05). Superoxide dismutase (SOD) activity was higher in the HP-H group compared with the HP-L group, but did not differ significantly between the HP-H and HP-M groups. Glutathione peroxidase (GSH-Px) activity was significantly higher in the HP-H group than in the HP-L group, whereas no significant difference was observed between the HP-H and HP-M groups. Conversely, malondialdehyde (MDA) concentration did not differ significantly among the three dietary treatments.

### 3.2. Digestive Enzyme Activity and Immune Indicators

As detailed in Table 3, the dietary N:S ratios significantly influenced ruminal digestive enzyme activities and immune parameters. Specifically, cellulase activity was significantly higher in the HP-H group compared with both the HP-M and HP-L groups (*p* < 0.05). In contrast, the activities of α-amylase, trypsin, and lipase were highest in the HP-M group, which were significantly greater than those in the HP-H and HP-L groups (*p* < 0.05). Chymotrypsin activity showed no significant differences among the dietary treatments (*p* > 0.05). Regarding humoral immune parameters, IgM concentrations were significantly lower in the HP-H group compared with the HP-M and HP-L groups (*p* < 0.05), whereas no significant differences were observed in IgA and IgG levels among the treatments (*p* > 0.05).

### 3.3. Volatile Fatty Acid Index

As detailed in Table 4, the dietary N:S ratio significantly affected ruminal fermentation parameters. The concentrations of propionate and butyrate were significantly higher in the HP-H group compared with the HP-M group, and butyrate was also significantly higher in the HP-H group than in the HP-L group (*p* < 0.05). In contrast, ammonia nitrogen, acetate, and valerate did not differ significantly among dietary treatments (*p* > 0.05), although acetate and valerate showed numerically higher values in the HP-H group.

### 3.4. Analysis of16S rRNA Gene Sequences of Rumen Microbiota

**Figure 1:**
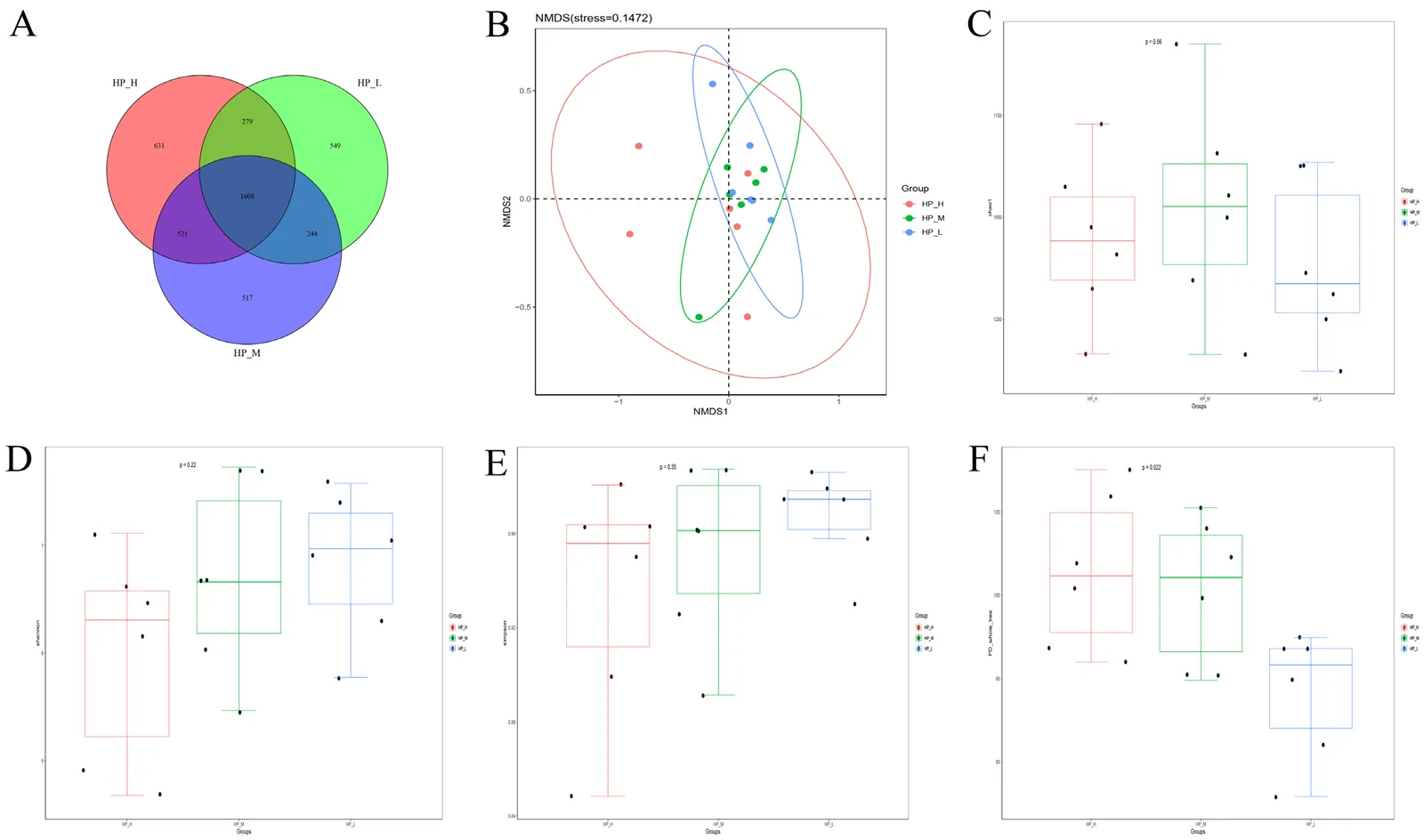
Venn diagram of rumen microbial diversity (**A**). NMDS principal component analysis diagram (**B**). Box plots of Chao1 index, Shannon index, Simpson index, and PD_whole_tree index (**C**–**F**). HP-H represents a nitrogen-to-sulfur ratio of 9.5:1. HP-M represents a nitrogen-to-sulfur ratio of 8.5:1. HP-L represents a nitrogen-to-sulfur ratio of 7.5:1.

As depicted in Figure 1A, a total of 4105 operational taxonomic units (OTUs) were identified across the three dietary treatments, with 631, 549, and 517 OTUs unique to the HP-H, HP-M, and HP-L groups, respectively. NMDS analysis (Figure 1B) demonstrated distinct separation among the dietary groups, reflecting substantial differences in the overall microbial community structure (Table S1). Regarding alpha diversity (Figure 1C–F), the PD whole-tree index was significantly reduced in the HP-L group compared to both the HP-H and HP-M treatments (*p* < 0.05). Additionally, the Chao1 and Shannon indices peaked in the HP-M group, whereas the Simpson index was numerically highest in the HP-H group.

**Figure 2:**
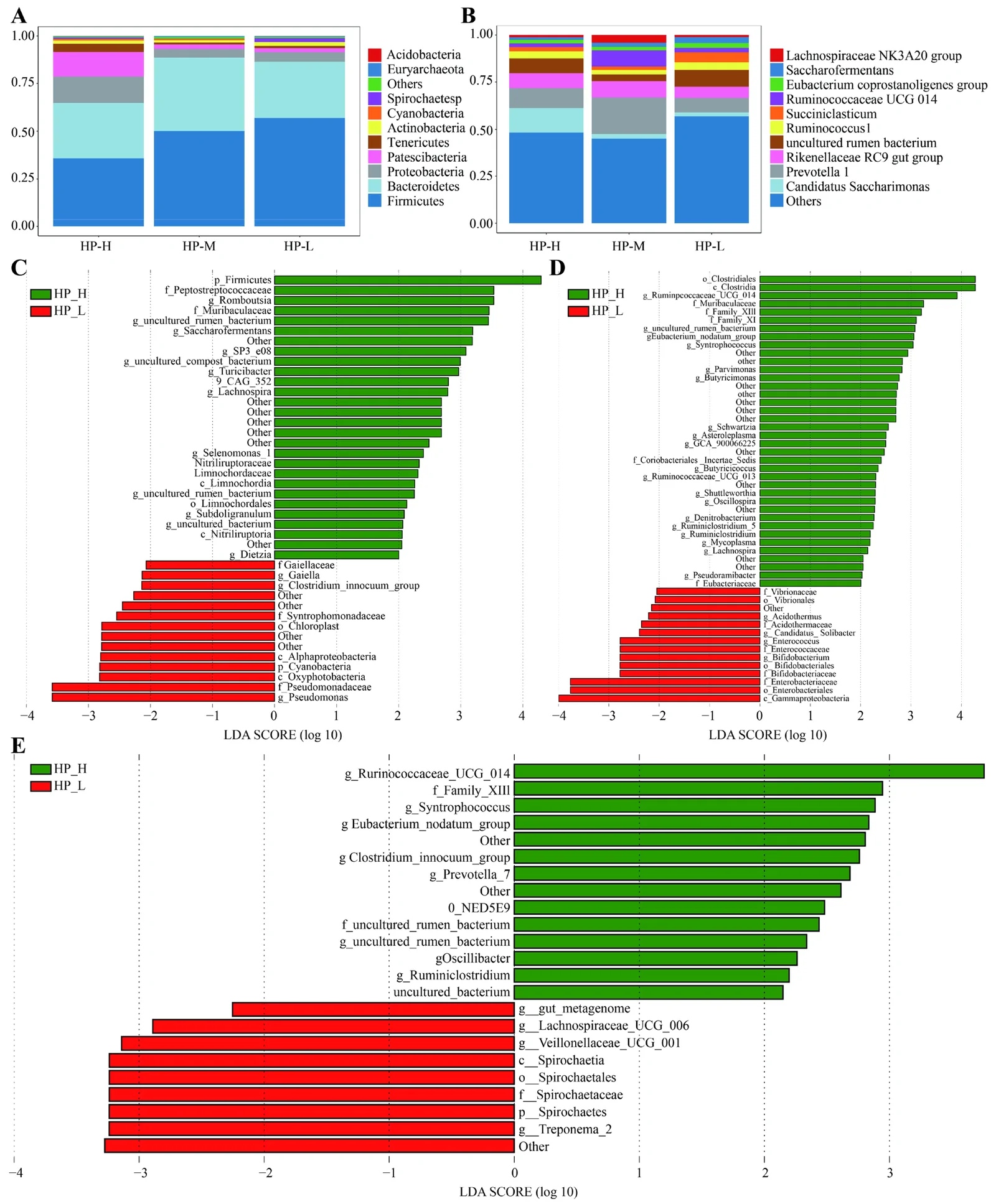
The profile and characteristics of the ruminal microbiota. Percentage of main colonies of rumen microorganisms (**A**). Percentage of main genera of rumen microorganisms (**B**). LDA analysis at the genus level (**C**–**E**). HP-H represents a nitrogen-to-sulfur ratio of 9.5:1. HP-M represents a nitrogen-to-sulfur ratio of 8.5:1. HP-L represents a nitrogen-to-sulfur ratio of 7.5:1.

Taxonomic composition analysis revealed that *Firmicutes, Bacteroidetes*, and *Proteobacteria* constituted the three dominant bacterial phyla across all treatments (Figure 2A). Numerically, the relative abundance of *Firmicutes* peaked in the HP-M group, whereas *Bacteroidetes* and *Proteobacteria* were most abundant in the HP-H group. At the genus level, *Candidatus Saccharimonas, Prevotella 1*, and *the Rikenellaceae RC9 gut group* predominated (Figure 2B). Specifically, *Prevotella 1* was maximized in the HP-M group, while *Candidatus Saccharimonas* and the *Rikenellaceae RC9 gut group* were most enriched in the HP-H treatment and exhibited a decreasing trend with lower N:S ratios. Furthermore, linear discriminant analysis effect size (LEfSe, LDA score > 3.0) was used to identify differential taxa among the treatments (Figure 2C–E). Conversely, the HP-M group was characterized by a higher relative abundance of *Syntrophococcus*, *SP3-e08*, and *Ruminococcaceae UCG-014*. Finally, the HP-L group showed higher relative abundance of *Romboutsia, Saccharofermentans, Lachnospiraceae UCG-006, Veillonellaceae UCG-001*, and *Treponema 2* (Table S2).

### 3.5. Rumen Non-Targeted Metabolomics Analysis

**Figure 3:**
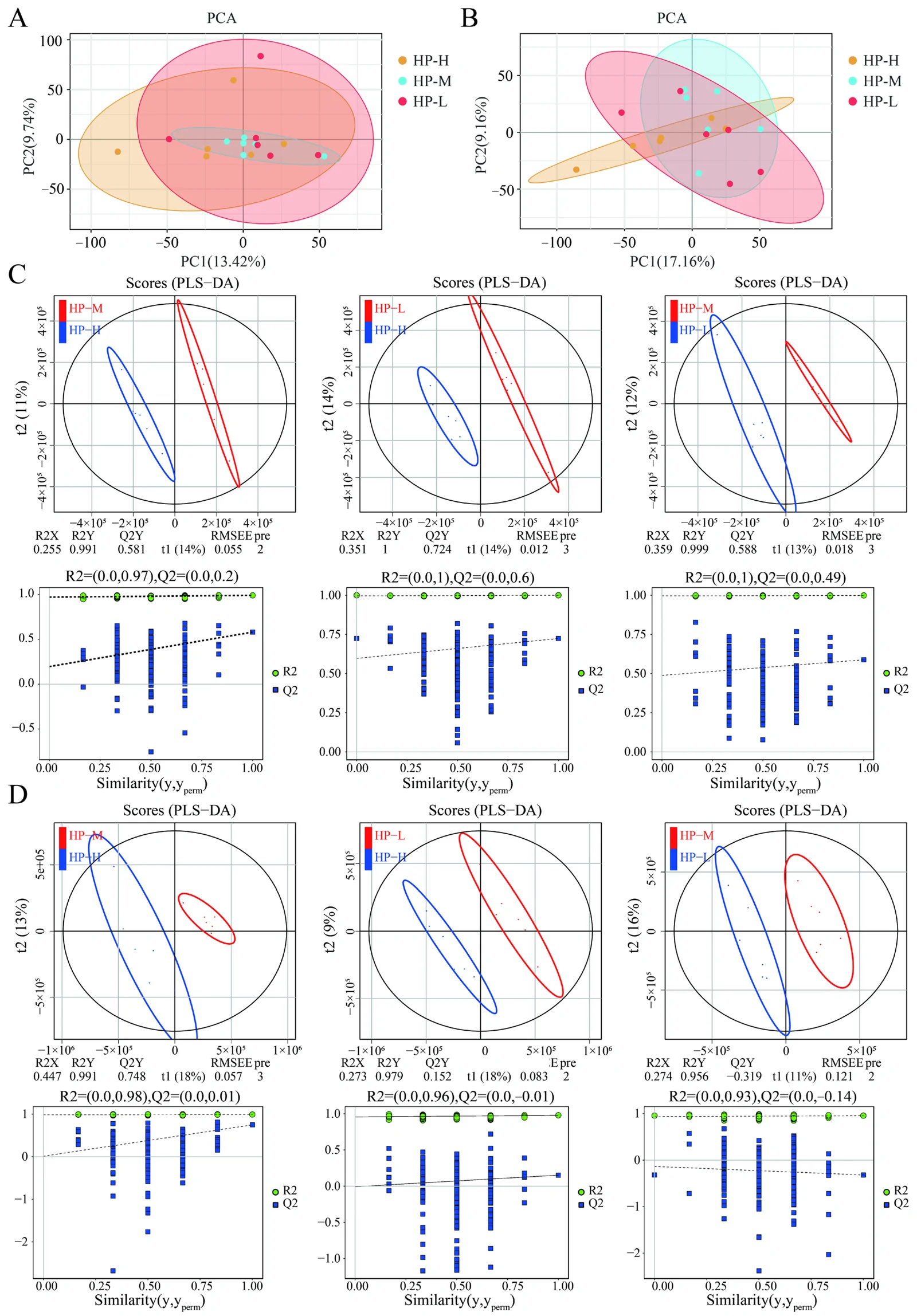
Score plot of principal component analysis (PCA) (**A**,**B**). Partial least squares discriminant analysis (PLS-DA) and permutation test plots among the three groups in positive ion mode (**C**). Partial least squares discriminant analysis (PLS-DA) and permutation test plots among the three groups in negative ion mode (**D**). HP-H represents a nitrogen-to-sulfur ratio of 9.5:1. HP-M represents a nitrogen-to-sulfur ratio of 8.5:1. HP-L represents a nitrogen-to-sulfur ratio of 7.5:1.

Principal component analysis (PCA) score plots in both positive and negative ion modes showed partial separation among the three dietary treatments (Figure 3A,B), indicating differences in metabolic profiles while also demonstrating a certain degree of overlap between groups. Orthogonal partial least squares discriminant analysis (OPLS-DA) was carried out to assess the metabolites in the samples. The R2Y value represents the cumulative variance explained by the model. The Q^2^ represents the proportion of variance in the data that is predicted by the current model. In positive ion mode (Figure 3C), the HP-H vs. HP-M and HP-H vs. HP-L comparisons yielded R^2^Y values of 0.993 and 0.991, with corresponding Q^2^ values of 0.549 and 0.587, respectively. In negative ion mode (Figure 3D), the HP-H vs. HP-M and HP-H vs. HP-L comparisons produced R^2^Y values of 0.993 and 0.995, with Q^2^ values of 0.752 and 0.543, respectively. In contrast, separation between the HP-M and HP-L groups was less pronounced, particularly in negative ion mode, where the model exhibited low predictive ability (R^2^Y = 0.953, Q^2^ = −0.096), suggesting limited predictive performance of the model for distinguishing these two dietary groups.

**Figure 4:**
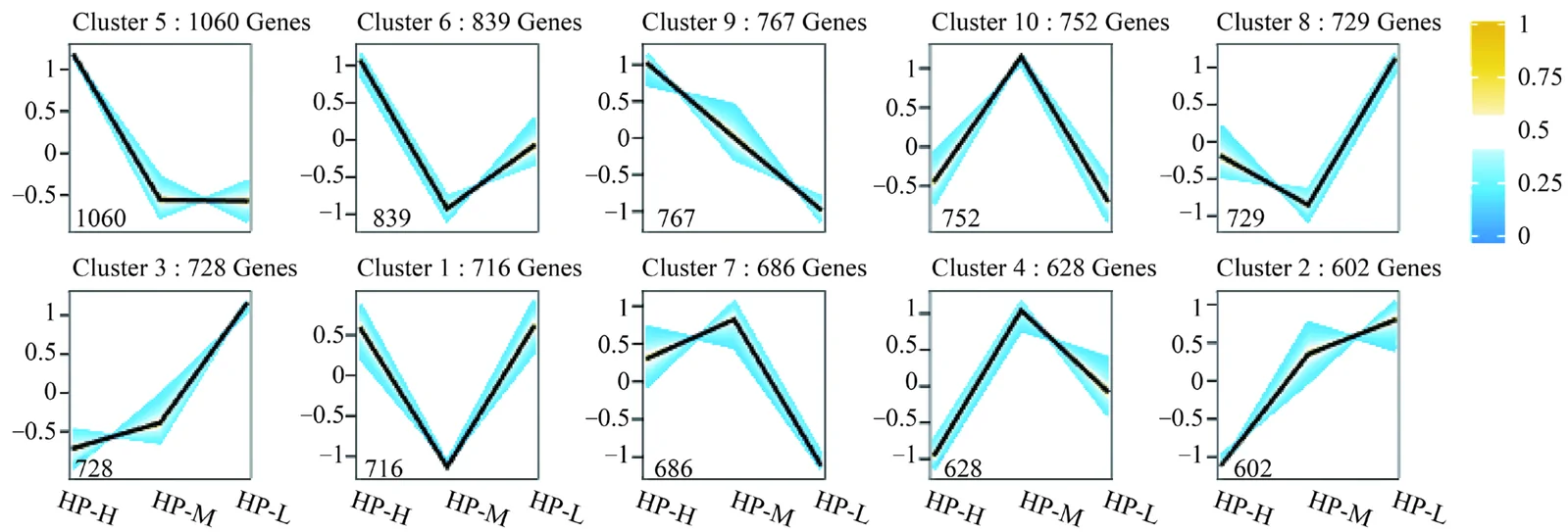
Metabolite expression level trend analysis chart. HP-H represents a nitrogen-to-sulfur ratio of 9.5:1. HP-M represents a nitrogen-to-sulfur ratio of 8.5:1. HP-L represents a nitrogen-to-sulfur ratio of 7.5:1.

We analyzed the expression patterns of all annotated metabolites (7507 in total) across different groups and characterized the variation trends in their abundance levels (Figure 4). After Mfuzz clustering analysis, 10 metabolite clusters were identified. Among them, Cluster 5 (1060 metabolites), Cluster 6 (839 metabolites), and Cluster 9 (767 metabolites) were the three most abundant clusters.

**Figure 5:**
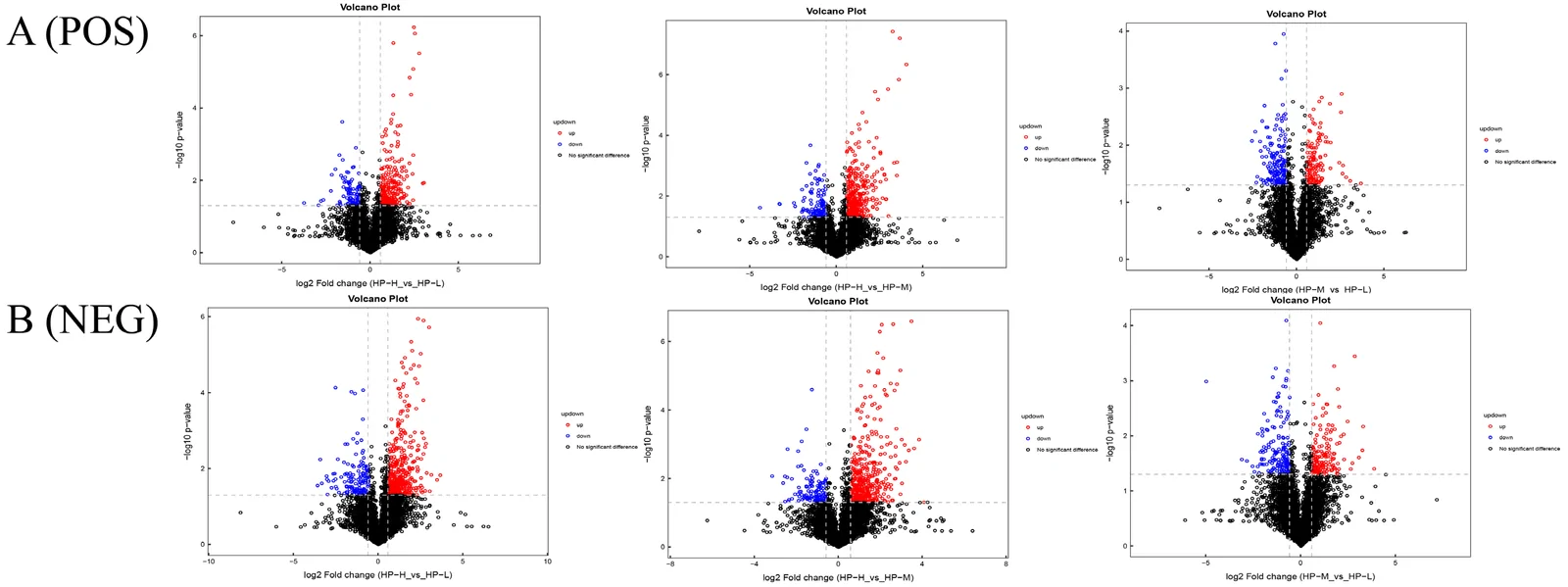
Differential metabolite analysis. Metabolic volcano plots among the three groups in the positive ion mode (**A**). Metabolic volcano plots among the three groups in the negative ion mode (**B**). HP-H represents a nitrogen-to-sulfur ratio of 9.5:1. HP-M represents a nitrogen-to-sulfur ratio of 8.5:1. HP-L represents a nitrogen-to-sulfur ratio of 7.5:1.

In the positive ion mode (Figure 5A), a total of 102 up-regulated and 41 down-regulated metabolites were identified in the HP-H vs. HP-L group, 141 up-regulated and 46 down-regulated metabolites in the HP-H vs. HP-M group, and 49 up-regulated and 71 down-regulated metabolites in the HP-M vs. HP-L group. In the negative ion mode (Figure 5B), 80 up-regulated and 28 down-regulated metabolites were identified in the HP-H vs. HP-L group, 68 up-regulated and 17 down-regulated metabolites in the HP-H vs. HP-M group, and 28 up-regulated and 38 down-regulated metabolites in the HP-M vs. HP-L group. Differential metabolites were determined based on peak intensity differences between experimental groups. Metabolites were selected using fold change (FC), variable importance in projection (VIP > 1), and *p* < 0.05. False discovery rate (FDR) correction was applied for multiple-testing control but was not used as a primary selection criterion (Table S3).

**Figure 6:**
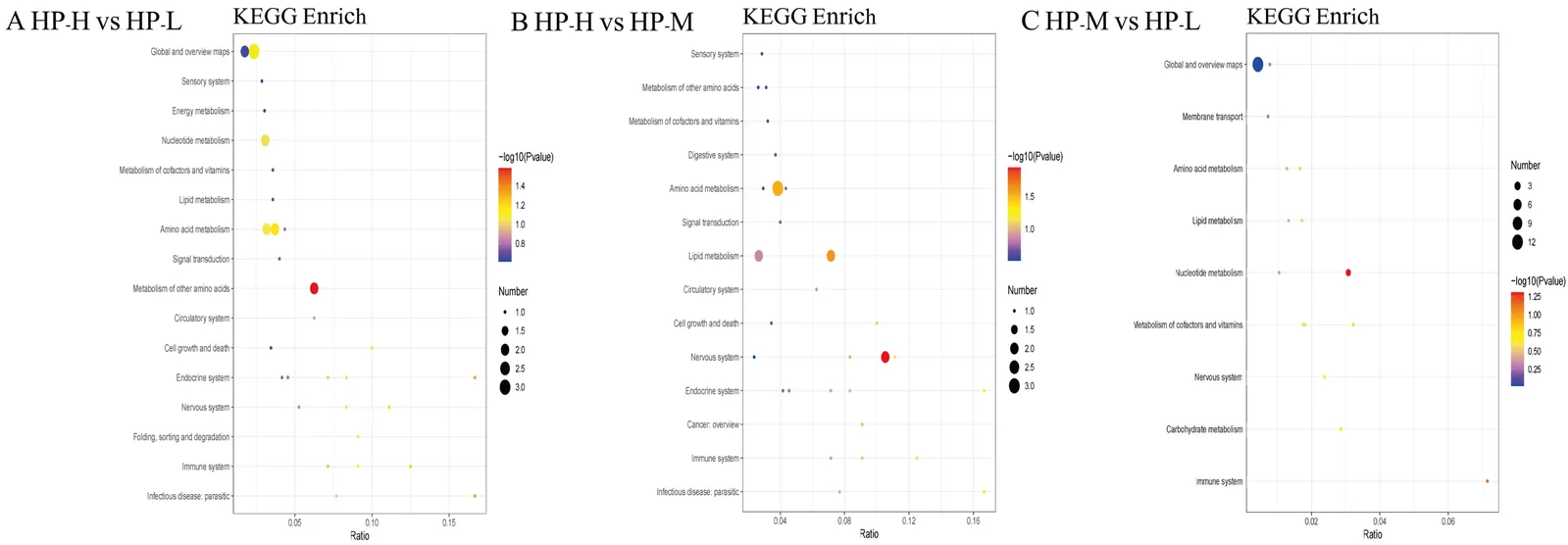
KEGG enrichment pathway analysis. HP-H vs. HP-L (**A**). HP-H vs. HP-M (**B**). HP-M vs. HP-L (**C**). HP-H represents a nitrogen-to-sulfur ratio of 9.5:1. HP-M represents a nitrogen-to-sulfur ratio of 8.5:1. HP-L represents a nitrogen-to-sulfur ratio of 7.5:1.

KEGG pathway enrichment analysis of differential metabolites revealed both shared and distinct metabolic perturbations among dietary treatments. Pathways showing enrichment trends based on nominal *p*-values were reported (Figure 6; Table S4). Overall, amino acid, lipid, cofactor and vitamin metabolism, along with KEGG-annotated nervous system-related pathways, showed consistent enrichment trends across comparisons. Differences between the HP-H and HP-L groups were mainly associated with trends in the biosynthesis of unsaturated fatty acids. Similarly, the HP-H vs. HP-M comparison showed similar enrichment trends, with additional involvement in the biosynthesis of unsaturated fatty acids.

**Figure 7:**
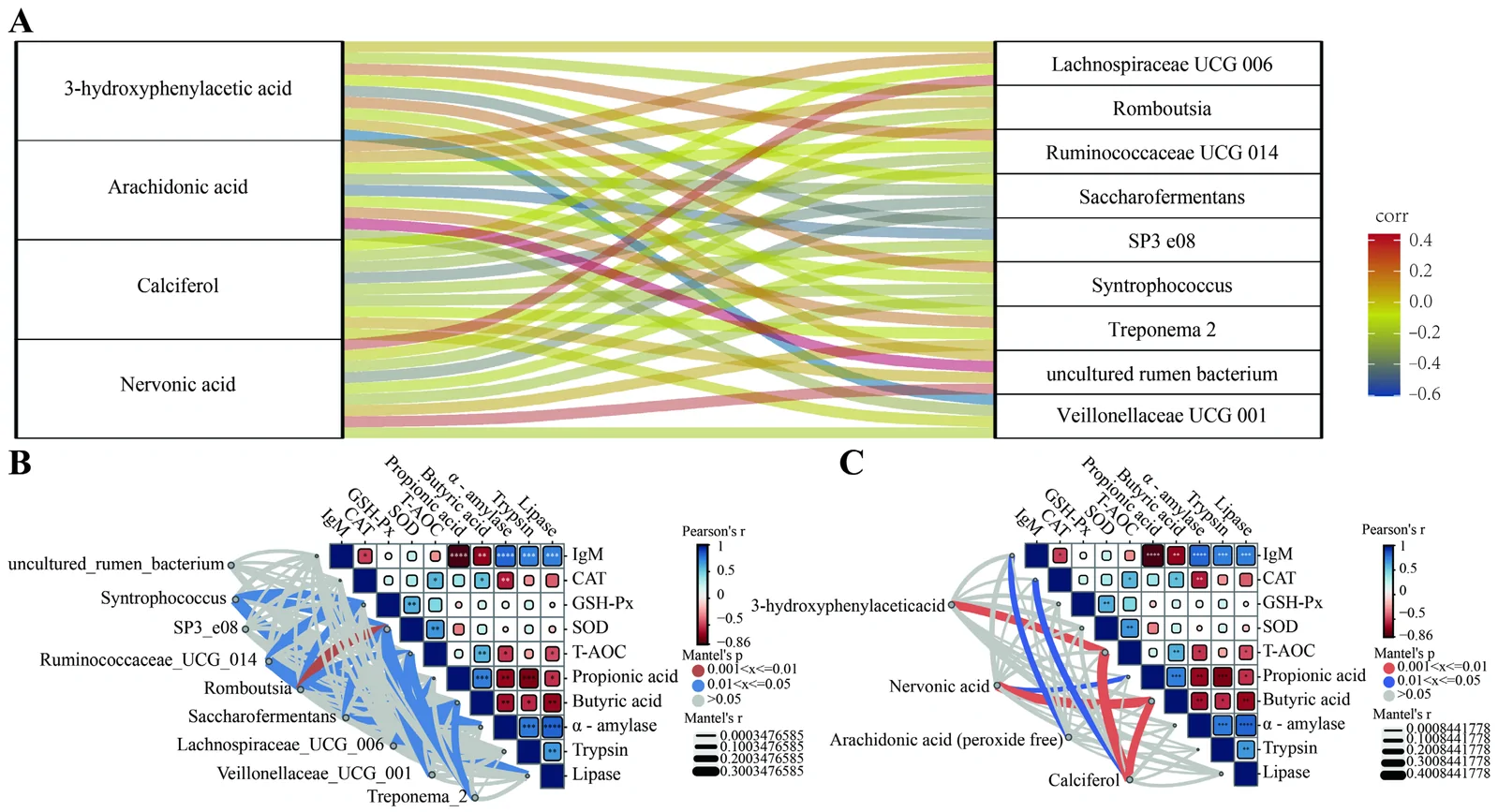
Correlation analysis. Sankey diagram between microorganisms and metabolites (**A**). Correlation analysis diagram between microorganisms and phenotypes (**B**). Correlation analysis diagram between metabolites and phenotypes (**C**). Significance levels are indicated as follows: * *p* < 0.05, ** *p* < 0.01, *** *p* < 0.001, and **** *p* < 0.0001.

To explore the potential relationships among differential metabolites, key microbial taxa, and rumen physiological characteristics, Pearson correlation analysis was performed. The Sankey diagram revealed an extensive association network between differential metabolites and key microbial taxa (Figure 7A). Among the identified metabolites, 3-hydroxyphenylacetic acid and arachidonic acid were significantly associated with multiple microbial taxa. Likewise, several bacterial genera, including *Lachnospiraceae UCG-006*, *Ruminococcaceae UCG-014*, and uncultured rumen bacterium, exhibited significant correlations with multiple differential metabolites. Further correlation analysis revealed significant associations between key microbial taxa and rumen functional parameters, including antioxidant capacity, digestive enzyme activities, and fermentation characteristics (Figure 7B). Notably, *Syntrophococcus* and *Ruminococcaceae UCG-014* were positively correlated with GSH-Px activity, as well as α-amylase and lipase activities. Similarly, correlation analysis between differential metabolites and rumen functional parameters revealed significant associations with antioxidant and fermentation-related traits (Figure 7C). Notably, calciferol was significantly positively correlated with CAT activity, whereas calciferol and 3-hydroxyphenylacetic acid were significantly negatively correlated with T-AOC activity. In addition, calciferol and nervonic acid exhibited significant negative correlations with butyrate concentration.

## 4. Discussion

The total antioxidant capacity (T-AOC) serves as a comprehensive indicator of the host’s redox status, primarily mediated by key antioxidant enzymes such as CAT, SOD, and GSH-Px [15]. GSH-Px mitigates oxidative damage by scavenging free radicals, while SOD catalyzes the dismutation of superoxide anions into oxygen and hydrogen peroxide, maintaining the delicate balance between oxidation and antioxidation [16,17]. In the present study, optimizing the N:S ratio to 9.5:1 was associated with higher ruminal antioxidant indices (i.e., T-AOC, CAT, SOD, and GSH-Px) under the conditions of this study.

Ruminal digestive enzyme activities directly govern dietary nutrient utilization and indirectly reflect the host’s capacity for nutrient acquisition [18]. Exogenous fibrolytic enzymes are known to enhance neutral detergent fiber (NDF) digestibility, particularly when endogenous fibrolytic activity is compromised by poor ruminal development or high-concentrate diets [19]. Ruminal cellulase activity was significantly higher at a N:S ratio of 9.5:1. Since ruminal digestive enzymes are primarily of microbial origin, this observation may be associated with changes in the ruminal microbial community structure and fermentation patterns. In contrast, the activities of α-amylase, trypsin, and lipase were lower at this ratio, accompanied by shifts in digestive enzyme activity profiles. Previous studies have indicated that dietary fiber components influence ruminal enzymatic activity. Therefore, these changes may reflect altered substrate utilization and microbial metabolic activity rather than direct regulation of enzyme secretion.

Volatile fatty acids (VFAs), the primary end-products of ruminal fermentation, fulfill 70–80% of the host’s energy requirements and serve as critical biomarkers for microbial fermentation efficiency [20,21]. Acetate, propionate, and butyrate constitute approximately 95% of total VFAs, exerting profound effects on ruminant metabolism [22]. By investigating the microbial community responses to host nutritional intake, the nitrogen in the intestine contributed to regulating the interaction between the gut microbiota and its host [23]. Dietary sulfur amino acid supplementation increased mucus thickness and the relative abundance of *Mucispirillum schaedleri*, thereby improving intestinal immune response [24]. In ruminants, short-chain fatty acids (SCFAs) stimulated sympathetic nervous system activation, thereby increasing energy expenditure and suppressing lipogenesis [25]. Pathway enrichment analysis revealed that butyrate mediates these effects by activating the Keap1-Nrf2 antioxidant pathway while suppressing NF-κB inflammatory signaling, thereby constructing a synergistic barrier defense network [26]. Our findings revealed that the total VFA, acetate, propionate, and butyrate relative abundance peaked at a N ratio of 9.5:1. Based on the established physiological roles of VFAs, particularly butyrate, it may be hypothesized that the metabolic environment generated under the 9.5:1 dietary N ratio was more favorable for maintaining ruminal epithelial homeostasis and immune stability. Interestingly, the lowest ruminal IgM concentration was also observed in this group. Although the biological significance of this observation remains to be fully elucidated, the concurrent increase in beneficial fermentation products and decrease in IgM may indicate a coordinated host–microbiota response associated with improved ruminal physiological status under the 9.5:1 N ratio.

Ruminal microbial diversity and richness parameters elucidate the modulatory effects of different N:S ratios on the microbiome. Alpha-diversity analysis revealed that a low N:S ratio (7.5:1) significantly reduced the PD whole-tree index, indicating a depletion in phylogenetic diversity. This may be attributed to an insufficient sulfur supply (e.g., restricted methionine precursors), which competitively inhibits sulfur-dependent microbial taxa, leading to community simplification. Taxonomic profiling confirmed that *Firmicutes, Bacteroidetes*, and *Proteobacteria* constituted the core microbiome in Tibetan sheep. *Bacteroidetes* primarily drive the degradation of complex plant polysaccharides, whereas *Firmicutes* participate in energy homeostasis via host-microbe metabolic crosstalk [27,28]. *Proteobacteria* contribute critically to VFA synthesis [5]. We observed that the relative abundances of Bacteroidetes and Proteobacteria peaked at a N:S ratio of 9.5:1. This taxonomic enrichment may be associated with carbohydrate degradation and VFA synthesis, ultimately improving host energy utilization. At the genus level, *Prevotella 1*, *the Rikenellaceae RC9 gut group*, and *Candidatus Saccharimonas* predominated. While *Prevotella* abundance peaked at 8.5:1, *Candidatus Saccharimonas* and the *Rikenellaceae RC9 gut group*, which are implicated in acetic acid-mediated lipid metabolism, were most enriched at 9.5:1, suggesting enhanced ruminal lipid metabolism at this ratio.

Untargeted metabolomics combined with multivariate analyses (PCA and PLS-DA) revealed distinct metabolic divergence driven by the dietary N:S ratios. Differential metabolites were predominantly mapped to amino acid, lipid, and coenzyme/vitamin metabolic pathways. Specifically, the relative abundance of 3-hydroxyphenylacetic acid, nervonic acid, arachidonic acid (peroxide-free), and calciferol was significantly elevated in the HP-H (9.5:1) group. 3-hydroxyphenylacetic acid is an intestinal derivative of dietary flavonoids, which possess potent antioxidant, anti-inflammatory, and methane-mitigating properties [29]. The ruminal biotransformation of flavonoids into phenolic acids is primarily driven by Bacteroidetes, Proteobacteria, and Firmicutes [30]. Consequently, the concurrent enrichment of Bacteroidetes and Proteobacteria at the 9.5:1 N:S ratio is likely associated with the elevated production of 3-hydroxyphenylacetic acid.

Calciferol (vitamin D_2_) is critical for maintaining calcium homeostasis, bone development, and immune function [31]. In high-altitude or low-sunlight environments, microbial synthesis of vitamin D_2_ can account for a substantial portion of the host’s daily requirements [32]. Anaerobic ruminal fungi and archaea convert plant-derived ergosterol into vitamin D_2_ via β-glucosidase-mediated hydrolysis—an enzyme primarily secreted by Bacteroidetes [33]. The increased abundance of Bacteroidetes at the 9.5:1 N:S ratio was correlated with the elevated relative abundance of calciferol, suggesting a potential association between microbial composition and vitamin-related metabolites.

Nervonic acid (NA), an ultra-long-chain monounsaturated fatty acid, has been reported to be associated with lipid metabolism, antioxidant processes, and gut microbiota-related metabolic regulation in mammals [34,35]. Arachidonic acid (ARA), an important polyunsaturated fatty acid, participates in multiple physiological processes through its metabolic pathways, including growth, immune regulation, stress response, lipid deposition, and skeletal development [36,37]. Moreover, the ARA metabolic pathway and its lipid derivatives have been suggested as potential targets involved in host–parasite interactions [38]. Notably, in the present study, a dietary nitrogen-to-sulfur ratio of 9.5:1 was associated with higher relative abundances of nervonic acid, arachidonic acid, and propionate compared with the other treatments. Nervonic acid showed a positive correlation with butyrate, suggesting a potential association between short-chain fatty acid profiles and long-chain lipid-related metabolites in the rumen environment. From a metabolic perspective, volatile fatty acids such as propionate may contribute to the overall acetyl-CoA pool, which is a central intermediate in lipid metabolism. However, in ruminants, the specific biochemical pathways linking individual VFAs to long-chain fatty acid variation remain complex and are likely influenced by multiple host–microbiota metabolic interactions. In this context, the observed enrichment of Bacteroidetes under the 9.5:1 N:S ratio may reflect enhanced carbohydrate fermentation capacity, which is closely associated with increased production of volatile fatty acids and overall rumen metabolic activity, thereby contributing to lipid metabolic homeostasis.

## 5. Conclusions

In conclusion, the dietary nitrogen-to-sulfur ratios influenced the ruminal antioxidant status of Tibetan sheep by modulating the ruminal microbiota involving *Candidatus Saccharimonas* and the *Ruminococcaceae RC9 gut group*, and differential metabolites (i.e., 3-hydroxyphenylacetic acid, nervonic acid, arachidonic acid and calciferol). These microbial and metabolic changes were associated with lipid metabolism and cofactor/vitamin metabolism pathways. The 9.5:1 dietary N/S ratio showed more favorable ruminal antioxidant and fermentation-related profiles among the tested treatments in Tibetan sheep.

## Figures and Tables

**Table 1:** Dietary concentrate composition and nutrient levels (dry matter basis).

Items	HP-H	HP-M	HP-L
Ingredient (%)			
Oat hay	15.00	15.00	15.00
Corn silage	15.00	15.00	15.00
Corn	32.41	32.41	32.41
Wheat	4.90	4.90	4.90
Soybean meal	5.60	5.60	5.60
Rapeseed meal	11.20	11.20	11.20
Cottonseed meal	1.40	1.40	1.40
Maize germ meal	1.40	1.40	1.40
Palm meal	8.40	8.40	8.40
Premix ^1^	2.94	2.94	2.94
NaCl	0.70	0.70	0.70
Baking soda	0.07	0.07	0.07
Limestone	0.70	0.70	0.70
Lysine	0.21	0.19	0.14
Methionine	0.07	0.09	0.14
Total	100	100	100
Nutrient levels			
DE (MJ·kg^−1^) ^2^	12.35	12.35	12.35
Crude protein %	12.73	12.73	12.73
Crude fat %	3.23	3.23	3.23
Neutral detergent fiber %	31.50	31.50	31.50
Acid detergent fiber %	20.14	20.14	20.14
Calcium %	0.51	0.51	0.51
Phosphorus %	0.18	0.18	0.18
Nitrogen %	2.163	2.081	2.012
Sulfur %	0.227	0.245	0.267
Nitrogen/Sulfur	9.5:1	8.5:1	7.5:1

^1^ Provided per kilogram of diets: Cu 15 mg, Fe 55 mg, Zn 25 mg, Mn 40 mg, Se 0.30 mg, I 0.5 mg, Co 0.20 mg, VA 20,000 IU, VD 4000 IU, VE 40 IU. ^2^ Digestive energy is the calculated value, while the rest are measured values. In the HP-H group, the nitrogen–sulfur ratio in the diet is 9.5:1. In the HP-M group, the nitrogen–sulfur ratio in the diet is 8.5:1. In the HP-L group, the nitrogen–sulfur ratio in the diet is 7.5:1.

**Table 2:** Determination of the antioxidant index.

Items	Groups			SEM	*p*-Value		
	HP-H	HP-M	HP-L		ANOVA	Linear	Quadratic
CAT (U/L)	200.41 ^a^	181.52 ^b^	183.05 ^b^	3.17	0.015	0.014	0.078
GSH-Px (U/mL)	946.80 ^a^	931.98 ^ab^	878.68 ^b^	11.59	0.030	0.012	0.368
MDA (nmol/mL)	15.42	14.88	16.05	0.24	0.126	0.259	0.085
SOD (U/mL)	183.89 ^a^	180.85 ^a^	154.85 ^b^	3.66	<0.001	<0.001	0.015
T-AOC (umol/mL)	60.44 ^a^	55.63 ^b^	53.26 ^b^	0.87	<0.001	<0.001	0.271

Note: ^a,b^ Means with different superscripts in the same row are significantly different (*p* < 0.05). Data are presented as mean ± SEM. SEM = standard error of the mean. CAT = Catalase. SOD = Superoxide dismutase. GSH-Px = Glutathione peroxidase. MDA = Malondialdehyde. T-AOC = Total antioxidant capacity.

**Table 3:** Determination of digestive enzyme activity and immune indicators.

Items	Groups			SEM	*p*-Value		
	HP-H	HP-M	HP-L		ANOVA	Linear	Quadratic
Digestive enzyme activity index							
α-amylase (U/mL)	144.01 ^c^	202.86 ^a^	168.76 ^b^	7.29	<0.001	0.045	<0.001
Chymotrypsin (U/mL)	174.24	168.94	175.00	3.32	0.743	0.930	0.451
Cellulase (U/mL)	368.66 ^a^	156.74 ^b^	142.33 ^b^	33.53	0.002	0.001	0.068
Trypsin (U/mL)	446.67 ^b^	568.04 ^a^	465.15 ^b^	17.73	0.003	0.567	0.001
Lipase (U/mL)	378.47 ^c^	631.80 ^a^	515.47 ^b^	32.07	0.001	0.019	0.001
Immune indicators							
IgA (mg/mL)	225.71	253.18	229.41	6.32	0.154	0.787	0.065
IgG (mg/mL)	620.65	596.57	627.13	31.81	0.937	0.944	0.735
IgM (mg/mL)	1249.09 ^b^	1529.39 ^a^	1296.06 ^b^	47.18	0.004	0.407	0.001

^a,b,c^ Means with different superscripts in the same row are significantly different (*p* < 0.05). Data are presented as mean ± SEM. SEM = standard error of the mean. IgA = immunoglobulin A. IgM = immunoglobulin M. IgG = immunoglobulin G.

**Table 4:** Determination of volatile fatty acid index.

Items	Groups			SEM	*p*-Value		
	HP-H	HP-M	HP-L		ANOVA	Linear	Quadratic
Ammoniacal nitrogen (mmol/L)	329.40	166.40	254.20	32.25	0.102	0.273	0.059
Propionic acid (mmol/L)	28.77 ^a^	19.30 ^b^	27.43 ^a^	1.66	0.009	0.560	0.003
Butyric acid (mmol/L)	12.33 ^a^	7.05 ^b^	8.88 ^b^	0.89	0.014	0.030	0.015
Valeric acid (mmol/L)	2.24	1.60	1.80	0.16	0.292	0.291	0.241
Acetic acid (mmol/L)	90.63	71.40	79.50	4.86	0.302	0.361	0.210

^a,b^ Means with different superscripts in the same row are significantly different (*p* < 0.05). Data are presented as mean ± SEM. SEM = standard error of the mean.

## Data Availability

The 16S rRNA gene sequencing data generated in this study are available in the NCBI Sequence Read Archive (SRA) under accession number PRJNA1472923 (released on 6 June 2026). The untargeted metabolomics data are available in the OMIX database of the China National Center for Bioinformation/Beijing Institute of Genomics, Chinese Academy of Sciences (https://ngdc.cncb.ac.cn/omix (accessed on 7 June 2026)) under accession number OMIX017330 (released on 6 June 2026).

## Supplementary Materials

The following supporting information can be downloaded at: https://www.mdpi.com/article/10.3390/antiox15080913/s1, Table S1. PERMANOVA (adonis) and ANOSIM analyses of bacterial beta diversity among dietary treatment groups. Table S2. Differential bacterial genera identified among pairwise comparisons of the three dietary treatment groups based on LEfSe analysis. Table S3. Statistical information for representative differential metabolites discussed in the manuscript. Table S4. Complete list of KEGG pathways enriched from differential metabolites.
