# Supplementary material for: Effect of Dietary Nitrogen-to-Sulfur Ratio on Rumen Microbiota, Metabolites, and Ruminal Antioxidant Status in Tibetan Sheep
Source: Antioxidants (Basel). 2026 Jul 23;15(8):913. doi: 10.3390/antiox15080913 (PMC13510054; doi:10.3390/antiox15080913)
Supplement: Supplementary file 1 [file antioxidants-15-00913-s001.zip › antioxidants-4379507-supplementary.pdf]

Table S1 PERMANOVA (adonis) and ANOSIM analyses of bacterial beta diversity among dietary treatment groups.

Items	PERMANOVA (R^2)	*P*-value	ANOSIM (R)	*P*-value
HP-M vs HP-L	0.127	0.018	0.200	0.024
HP-H vs HP-L	0.130	0.070	0.181	0.059
HP-H vs HP-M vs HP-L	0.163	0.015	0.176	0.006

Table S2 Differential bacterial genera identified among pairwise comparisons of the three dietary treatment groups based on LEfSe analysis.

Microorganism	HP-H	HP-L	HP-M
HP-H VS HP-M			
Acidothermus	7.17	3.33	0.50
Candidatus_Solibacter	6.00	2.50	0.00
Enterococcus	402.33	99.17	57.83
Bifidobacterium	423.33	152.17	51.33
Ruminococcaceae_UCG_014	1266.33	1118.67	5215.50
uncultured_rumen_bacterium	4925.17	4537.33	2321.50
Eubacterium__nodatum_group	0.50	0.17	5.67
Syntrophococcus	81.83	75.00	599.50
Parvimonas	0.17	0.83	1.33
Butyricimonas	0.33	0.33	1.83
Schwartzia	0.83	31.83	14.00
Asteroleplasma	2.50	3.00	6.83
GCA_900066225	0.50	0.67	3.33
Butyricicoccus	1.83	27.17	18.17
Ruminococcaceae_UCG_013	78.17	101.17	199.7
Shuttleworthia	4.67	10.17	26.00
Oscillospira	0.83	6.00	11.67
Denitrobacterium	3.33	10.50	13.83
Ruminiclostridium_5	4.17	11.00	14.00
Ruminiclostridium	20.17	21.50	103.00
Mycoplasma	1.50	18.17	12.50
Lachnospira	2.67	225.50	18.00
Pseudoramibacter	6.50	22.67	21.50
HP-H VS HP-L			
Gaiella	5.17	0.00	0.00
Clostridium__innocuum_group	0.00	0.00	0.00
Pseudomonas	2751.50	306.67	391.17
Romboutsia	96.67	1639.50	86.17
uncultured_rumen_bacterium	4925.17	4537.33	2321.50
Saccharofermentans	840.67	1490.67	1308.50

SP3_e08	86.17	719.17	727.83
uncultured_compost_bacterium	0.33	3.00	0.83
Turicibacter	3.67	424.83	13.67
CAG_352	9.83	363.83	11.17
Lachnospira	2.67	225.50	18.00
Selenomonas_1	34.33	151.17	154.50
uncultured_rumen_bacterium	4925.17	4537.33	2321.50
Subdoligranulum	25.33	71.33	111.67
uncultured_bacterium	2614.50	394.00	721.50
Dietzia	1.17	3.17	0.50
HP-M VS HP-L			
gut_metagenome	1.67	14.67	7.17
Lachnospiraceae_UCG_006	4.83	5.33	3.67
Veillonellaceae_UCG_001	249.00	728.33	197.83
Treponema_2	419.67	1315.00	100.83
Ruminococcaceae_UCG_014	1266.33	1118.67	5215.50
Syntrophococcus	81.83	75.00	599.50
Eubacterium__nodatum_group	0.50	0.17	5.67
Clostridium__innocuum_group	0.00	0.00	0.00
Prevotella_7	142.67	73.83	356.17
uncultured_rumen_bacterium	4925.17	4537.33	2321.50
Oscillibacter	5.50	3.17	12.50
Ruminiclostridium	20.17	21.50	103.00
uncultured_bacterium	2614.50	394.00	721.50

Table S3 Statistical information for representative differential metabolites discussed in the manuscript.

Name	VIP	Fold change	p-value	m/z	rt(s)	FDR
Nervonic acid	2.783617387	2.090408924	1.97024E-05	365.34215	69.94525	0.000118
Arachidonic acid (peroxide free)	1.433156925	2.526744568	0.001743753	311.25913	85.59215	0.005232
Calciferol	3.495620349	3.434430043	0.042737065	397.35797	49.8826	0.064105
alpha-Tocopherol (Vitamin E)	0.190982014	1.430848635	0.219842779	429.37336	48.391	0.219843
Pyridoxamine	2.370524096	1.763025352	0.066324781	169.09747	129.085	0.07959
3-hydroxyphenylaceti	1.17040038	1.43020886	0.01019047	107.0491	57.7635	0.02038

c acid	2	8	3	7

Table S4 Complete list of KEGG pathways enriched from differential metabolites

Pathway-Hierarchy1	Pathway-Hierarchy2	Map-ID	Map-Name	p-value	FDR	richFactor
HP-H vs HP-L						
Metabolism	Lipid metabolism	oas01040	Biosynthesis of unsaturated fatty acids	0.000	0.012	0.068
Metabolism	Metabolism of other amino acids	oas00410	beta-Alanine metabolism	0.026	0.276	0.063
Organismal Systems	Endocrine system	oas04912	GnRH signaling pathway	0.047	0.276	0.167
Human Diseases	Infectious disease: parasitic	oas05140	Leishmaniasis	0.047	0.276	0.167
Organismal Systems	Immune system	oas04666	Fc gamma R-mediated phagocytosis	0.062	0.276	0.125
Metabolism	Amino acid metabolism	oas00310	Lysine degradation	0.068	0.276	0.037
Organismal Systems	Nervous system	oas04730	Long-term depression	0.069	0.276	0.111
Cellular Processes	Cell growth and death	oas04217	Necroptosis	0.077	0.276	0.100
Metabolism	Global and overview maps	oas01230	Biosynthesis of amino acids	0.079	0.276	0.023
Organismal Systems	Immune system	oas04664	Fc epsilon RI signaling pathway	0.084	0.276	0.091
Genetic Information Processing	Folding, sorting and degradation	oas04122	Sulfur relay system	0.084	0.276	0.091
Metabolism	Amino acid metabolism	oas00270	Cysteine and methionine metabolism	0.088	0.276	0.032
Organismal Systems	Endocrine system	oas04921	Oxytocin signaling pathway	0.091	0.276	0.083
Organismal Systems	Nervous system	oas04728	Dopaminergic synapse	0.091	0.276	0.083
Metabolism	Nucleotide metabolism	oas00240	Pyrimidine metabolism	0.093	0.276	0.031
Human Diseases	Infectious disease: parasitic	oas05146	Amoebiasis	0.098	0.276	0.077
Organismal Systems	Immune system	oas04611	Platelet activation	0.106	0.276	0.071
Organismal Systems	Endocrine system	oas04923	Regulation of lipolysis in adipocytes	0.106	0.276	0.071
Organismal Systems	Circulatory system	oas04270	Vascular smooth muscle contraction	0.120	0.296	0.063
Organismal Systems	Nervous system	oas04723	Retrograde endocannabinoid signaling	0.141	0.331	0.053
Organismal Systems	Endocrine system	oas04925	Aldosterone synthesis and	0.161	0.354	0.045

			secretion			
Metabolism	Amino acid metabolism	oas00290	Valine, leucine and isoleucine biosynthesis	0.168	0.354	0.043
Organismal Systems	Endocrine system	oas04913	Ovarian steroidogenesis	0.174	0.354	0.042
Environmental Information Processing	Signal transduction	oas04024	cAMP signaling pathway	0.181	0.354	0.040
Metabolism	Lipid metabolism	oas00591	Linoleic acid metabolism	0.200	0.360	0.036
Metabolism	Metabolism of cofactors and vitamins	oas00750	Vitamin B6 metabolism	0.200	0.360	0.036
Cellular Processes	Cell growth and death	oas04216	Ferroptosis	0.207	0.360	0.034
Metabolism	Global and overview maps	oas01200	Carbon metabolism	0.228	0.376	0.018
Metabolism	Energy metabolism	oas00920	Sulfur metabolism	0.232	0.376	0.030
Organismal Systems	Sensory system	oas04750	Inflammatory mediator regulation of TRP channels	0.244	0.377	0.029
Metabolism	Metabolism of other amino acids	oas00480	Glutathione metabolism	0.262	0.377	0.026
Organismal Systems	Digestive system	oas04977	Vitamin digestion and absorption	0.268	0.377	0.026
Organismal Systems	Nervous system	oas04726	Serotonergic synapse	0.285	0.377	0.024
Metabolism	Amino acid metabolism	oas00280	Valine, leucine and isoleucine degradation	0.285	0.377	0.024
Metabolism	Global and overview maps	oas01210	2-Oxocarboxylic acid metabolism	0.288	0.377	0.015
Environmental Information Processing	Membrane transport	oas02010	ABC transporters	0.297	0.377	0.015
Metabolism	Lipid metabolism	oas00592	alpha-Linolenic acid metabolism	0.297	0.377	0.023
Metabolism	Carbohydrate metabolism	oas00640	Propanoate metabolism	0.319	0.395	0.021
Metabolism	Metabolism of cofactors and vitamins	oas00790	Folate biosynthesis	0.367	0.426	0.018
Metabolism	Lipid metabolism	oas00061	Fatty acid biosynthesis	0.372	0.426	0.017
Metabolism	Lipid metabolism	oas00100	Steroid biosynthesis	0.372	0.426	0.017
Metabolism	Lipid metabolism	oas00590	Arachidonic acid metabolism	0.453	0.498	0.013
Metabolism	Amino acid metabolism	oas00330	Arginine and proline metabolism	0.466	0.498	0.013
Metabolism	Amino acid metabolism	oas00350	Tyrosine metabolism	0.466	0.498	0.013
Metabolism	Xenobiotics	oas00982	Drug metabolism -	0.504	0.526	0.011

	biodegradation and metabolism		cytochrome P450			
Organismal Systems	Digestive system	oas04976	Bile secretion	0.542	0.554	0.010
Metabolism	Global and overview maps	oas01100	Metabolic pathways	0.999	0.999	0.005
HP-H vs HP-M						
Metabolism	Lipid metabolism	oas01040	Biosynthesis of unsaturated fatty acids	0.004	0.163	0.054
Organismal Systems	Nervous system	oas04723	Retrograde endocannabinoid signaling	0.011	0.261	0.105
Metabolism	Lipid metabolism	oas00591	Linoleic acid metabolism	0.024	0.330	0.071
Metabolism	Amino acid metabolism	oas00350	Tyrosine metabolism	0.029	0.330	0.038
Organismal Systems	Endocrine system	oas04912	GnRH signaling pathway	0.051	0.330	0.167
Human Diseases	Infectious disease: parasitic	oas05140	Leishmaniasis	0.051	0.330	0.167
Organismal Systems	Immune system	oas04666	Fc gamma R-mediated phagocytosis	0.067	0.330	0.125
Organismal Systems	Nervous system	oas04730	Long-term depression	0.075	0.330	0.111
Cellular Processes	Cell growth and death	oas04217	Necroptosis	0.083	0.330	0.100
Organismal Systems	Immune system	oas04664	Fc epsilon RI signaling pathway	0.091	0.330	0.091
Human Diseases	Cancer: overview	oas05231	Choline metabolism in cancer	0.091	0.330	0.091
Organismal Systems	Endocrine system	oas04921	Oxytocin signaling pathway	0.099	0.330	0.083
Organismal Systems	Nervous system	oas04728	Dopaminergic synapse	0.099	0.330	0.083
Human Diseases	Infectious disease: parasitic	oas05146	Amoebiasis	0.107	0.330	0.077
Organismal Systems	Immune system	oas04611	Platelet activation	0.115	0.330	0.071
Organismal Systems	Endocrine system	oas04923	Regulation of lipolysis in adipocytes	0.115	0.330	0.071
Organismal Systems	Circulatory system	oas04270	Vascular smooth muscle contraction	0.130	0.349	0.063
Metabolism	Lipid metabolism	oas00590	Arachidonic acid metabolism	0.137	0.349	0.027
Organismal Systems	Endocrine system	oas04925	Aldosterone synthesis and secretion	0.174	0.409	0.045
Metabolism	Amino acid metabolism	oas00220	Arginine biosynthesis	0.182	0.409	0.043
Organismal Systems	Endocrine system	oas04913	Ovarian steroidogenesis	0.189	0.409	0.042
Environmental Information	Signal transduction	oas04024	cAMP signaling pathway	0.196	0.409	0.040

Processing						
Organismal Systems	Digestive system	oas04973	Carbohydrate digestion and absorption	0.210	0.420	0.037
Cellular Processes	Cell growth and death	oas04216	Ferroptosis	0.223	0.428	0.034
Metabolism	Metabolism of cofactors and vitamins	oas00730	Thiamine metabolism	0.237	0.431	0.032
Metabolism	Metabolism of other amino acids	oas00410	beta-Alanine metabolism	0.244	0.431	0.031
Metabolism	Amino acid metabolism	oas00400	Phenylalanine, tyrosine and tryptophan biosynthesis	0.257	0.432	0.029
Organismal Systems	Sensory system	oas04750	Inflammatory mediator regulation of TRP channels	0.263	0.432	0.029
Metabolism	Metabolism of other amino acids	oas00480	Glutathione metabolism	0.282	0.443	0.026
Organismal Systems	Nervous system	oas04726	Serotonergic synapse	0.307	0.443	0.024
Metabolism	Carbohydrate metabolism	oas00650	Butanoate metabolism	0.307	0.443	0.024
Metabolism	Lipid metabolism	oas00592	alpha-Linolenic acid metabolism	0.319	0.443	0.023
Metabolism	Metabolism of terpenoids and polyketides	oas00900	Terpenoid backbone biosynthesis	0.325	0.443	0.022
Environmental Information Processing	Membrane transport	oas02010	ABC transporters	0.334	0.443	0.015
Organismal Systems	Digestive system	oas04974	Protein digestion and absorption	0.337	0.443	0.021
Metabolism	Amino acid metabolism	oas00260	Glycine, serine and threonine metabolism	0.354	0.453	0.020
Metabolism	Lipid metabolism	oas00564	Glycerophospholipid metabolism	0.365	0.454	0.019
Metabolism	Lipid metabolism	oas00100	Steroid biosynthesis	0.398	0.482	0.017
Metabolism	Amino acid metabolism	oas00360	Phenylalanine metabolism	0.409	0.482	0.017
Metabolism	Amino acid metabolism	oas00330	Arginine and proline metabolism	0.496	0.570	0.013
Metabolism	Xenobiotics biodegradation and metabolism	oas00982	Drug metabolism - cytochrome P450	0.534	0.599	0.011
Metabolism	Metabolism of cofactors and vitamins	oas00130	Ubiquinone and other terpenoid-quinone biosynthesis	0.554	0.607	0.011

Organismal Systems	Digestive system	oas04976	Bile secretion	0.574	0.614	0.010
Metabolism	Global and overview maps	oas01230	Biosynthesis of amino acids	0.677	0.708	0.008
Metabolism	Global and overview maps	oas01210	2-Oxocarboxylic acid metabolism	0.694	0.709	0.007
Metabolism	Global and overview maps	oas01100	Metabolic pathways	0.995	0.995	0.006
HP-M vs HP-L						
Metabolism	Amino acid metabolism	oas00400	Phenylalanine, tyrosine and tryptophan biosynthesis	0.015	0.236	0.059
Metabolism	Nucleotide metabolism	oas00240	Pyrimidine metabolism	0.049	0.394	0.031
Organismal Systems	Immune system	oas04611	Platelet activation	0.075	0.399	0.071
Metabolism	Metabolism of cofactors and vitamins	oas00730	Thiamine metabolism	0.158	0.456	0.032
Metabolism	Carbohydrate metabolism	oas00030	Pentose phosphate pathway	0.177	0.456	0.029
Organismal Systems	Nervous system	oas04726	Serotonergic synapse	0.209	0.456	0.024
Metabolism	Metabolism of cofactors and vitamins	oas00760	Nicotinate and nicotinamide metabolism	0.264	0.456	0.018
Metabolism	Metabolism of cofactors and vitamins	oas00790	Folate biosynthesis	0.273	0.456	0.018
Metabolism	Lipid metabolism	oas00061	Fatty acid biosynthesis	0.277	0.456	0.017
Metabolism	Amino acid metabolism	oas00360	Phenylalanine metabolism	0.285	0.456	0.017
Metabolism	Lipid metabolism	oas00590	Arachidonic acid metabolism	0.343	0.472	0.013
Metabolism	Amino acid metabolism	oas00350	Tyrosine metabolism	0.354	0.472	0.013
Metabolism	Nucleotide metabolism	oas00230	Purine metabolism	0.413	0.508	0.011
Metabolism	Global and overview maps	oas01230	Biosynthesis of amino acids	0.514	0.574	0.008
Environmental Information Processing	Membrane transport	oas02010	ABC transporters	0.538	0.574	0.007
Metabolism	Global and overview maps	oas01100	Metabolic pathways	0.950	0.950	0.004
